# Absolute quantitation of post-translational modifications

**DOI:** 10.3389/fchem.2014.00058

**Published:** 2014-08-05

**Authors:** Ruchi Chaube

**Affiliations:** Department of Medicine and Institute for Transformative Molecular Medicine, Case Western Reserve University School of Medicine and University HospitalsCleveland, OH, USA

**Keywords:** absolute quantitation, Site-stoichiometry, Cys-based modifications, phosphorylation, stable isotope labeling, SILAC, iTRAQ, TMT

The cell's fate is largely governed by the dynamics of cellular events, brought about by the interplay of various proteins, in which post-translational modifications (PTMs) play a key role. Therefore, a better understanding of post-translational modification is of utmost importance to a biochemist. Over the years much work in this regard has been achieved through mass spectrometry, however, the “on-off” nature of PTMs, which accounts for cell's dynamics, has posed a serious challenge in quantitating them, especially with regards to measuring the occupancy of PTMs on a particular site. Protein phosphorylation has served as the best model for which measurement of occupancy has been attempted by employing stable isotope labeling and label-free approaches. But the reliability of these methods has mainly been limited to the relative quantitation between samples. Although the fractional occupancy/absolute quantitation of phosphorylation has been determined by grossly applying these methods, but overall they fall short of testability. Moreover, the field demands a broad approach which can even encompass a more challenging task of absolute quantitation of Cys-based PTMs.

For quantitation, differential labeling of samples with stable isotopes or in the label-free approach the peak intensity of a peptide ion or identification frequency of the peptides of a particular protein is used for measurement. Stable isotope labeling is achieved through metabolic labeling in cell culture or by chemical labeling in post-metabolic stage. For the metabolic labeling of proteins, the cell cultures are grown in the presence of naturally abundant amino acids (light version), and amino acids (primarily Leu, Lys, Arg) labeled with heavy isotopes (13C, 15N and/or deuterium), this method is known as stable isotope labeling with amino acids in cell culture (SILAC). The ratio of the ion intensities of the peptide (light vs. heavy) in the MS scan is used for quantitation (Ong et al., 2002). In the post-metabolic labeling approach, such as isotope-coded affinity tag (ICAT) (Sethuraman et al., 2004), isobaric tag for relative and absolute quantitation (iTRAQ) (Gan et al., 2007) and tandem mass tag (TMT) (Thompson et al., 2003), the peptide fragments generated by proteolytic digestion of proteins are chemically derivatized at an amino acid side chain; cysteines thiols (ICAT) or primary amines (iTRAQ and TMT) and quantitation of peptides (and hence the protein) between the samples is done by comparing the ion intensities of the tags in the MS/MS spectrum. Analogous to the SILAC, another approach called Stable isotope labeling in mammals (SILAM) was developed to introduce 15N-label rich diet into a mouse. The ratio of the ion intensities of 14N (light)/15N (heavy) peptides was used to check the changes in the protein expression in tissues (Wu et al., 2004; McClatchy et al., 2007), differential regulation of phosphorylation site within a protein (Tao et al., 2009) or in quantitative phosphoproteomics (Ficarro et al., 2002). The rationale behind SILAC and SILAM approaches is to counteract the potential problem of systematic errors associated in post-metabolic labeling strategy. Here, the light and heavy samples are mixed after the sample preparation, wherein, in the metabolic labeling approach, the translational machinery of the cell incorporates heavy label into the whole proteome, and the light and heavy samples are mixed prior to sample preparation.

Stable isotope labeling for absolute quantitation requires internal standards that are used to measure protein abundance *per se*. These standards are the pre-selected synthetic peptides from the protein of interest that gets labeled with an isotope. A known amount of the isotope labeled peptides are mixed with the sample and then introduced into the mass spectrometer. Absolute abundance of the PTM can be obtained by measuring the relative abundance of the modified and unmodified peptides and comparing it with the amount of standard used. Notably, stable isotope labeling and synthetic peptide standards were used in absolute quantitation of phosphorylation on Ser1526 in human separase (Stemmann et al., 2001) and phosphorylation in Akt (Atrih et al., 2010). Moreover, the SILAC approach was used to quantitate global phosphoproteomics during mitosis (Olsen et al., 2010). These methods have set the base for absolute quantitation, but they are inefficient and error-prone. The stable isotope labeling approach is biased, and the synthesis of peptide standards is costly, time-intensive and, may induce run-to-run variation in LC-MS/MS analysis. Also, the use of internal standards becomes unreliable when a pathophysiologic condition gives rise to a (new) site which is not included in the internal standards. Label-free approach is another way to quantify but in this technique, there is a common assumption that the ion intensities of the modified and unmodified peptides (peptides derived from the same protein) are the same which probably is not the case. Therefore, we need a better approach which can circumvent the shortcomings of the synthesized standards and address the issue of different ion intensities of modified and unmodified peptides.

Interestingly, an integrated phosphatase treatment and stable isotope labeling approach was adapted for determining the absolute phosphorylation occupancy in the yeast proteome. This approach did not make use of any standard, instead equal amounts of protein lysates after protease-digestion were either mock- or phosphatase treated, differentially labeled with stable isotope and subsequently mixed. The absolute phosphorylation site occupancy was represented by the single ratio between the non-phosphorylated peptides with and without phosphatase treatment (Wu et al., 2011). Another excellent approach; super-SILAC, that was originally proposed in Geiger et al. (2011), made use of SILAC only as a spike-in standard, making use of heavy labeled protein/s or proteome as a reference standard. The SILAC mixture was generated separately from the unlabeled test samples and was added to the test samples followed by protease digestion. Quantitation in this case is done by measuring the ion intensities and calculating the “ratio of ratios” as shown in Figure 1. Quantitation by this method eliminates the experimental variations brought by the synthetic peptides, because the reference protein is digested and processed along with the test sample. Recently, for the absolute quantitation of phosphoproteome in the cell cycle of fission yeast, the super-SILAC approach was elegantly used (Carpy et al., 2014).

**Figure 1:**
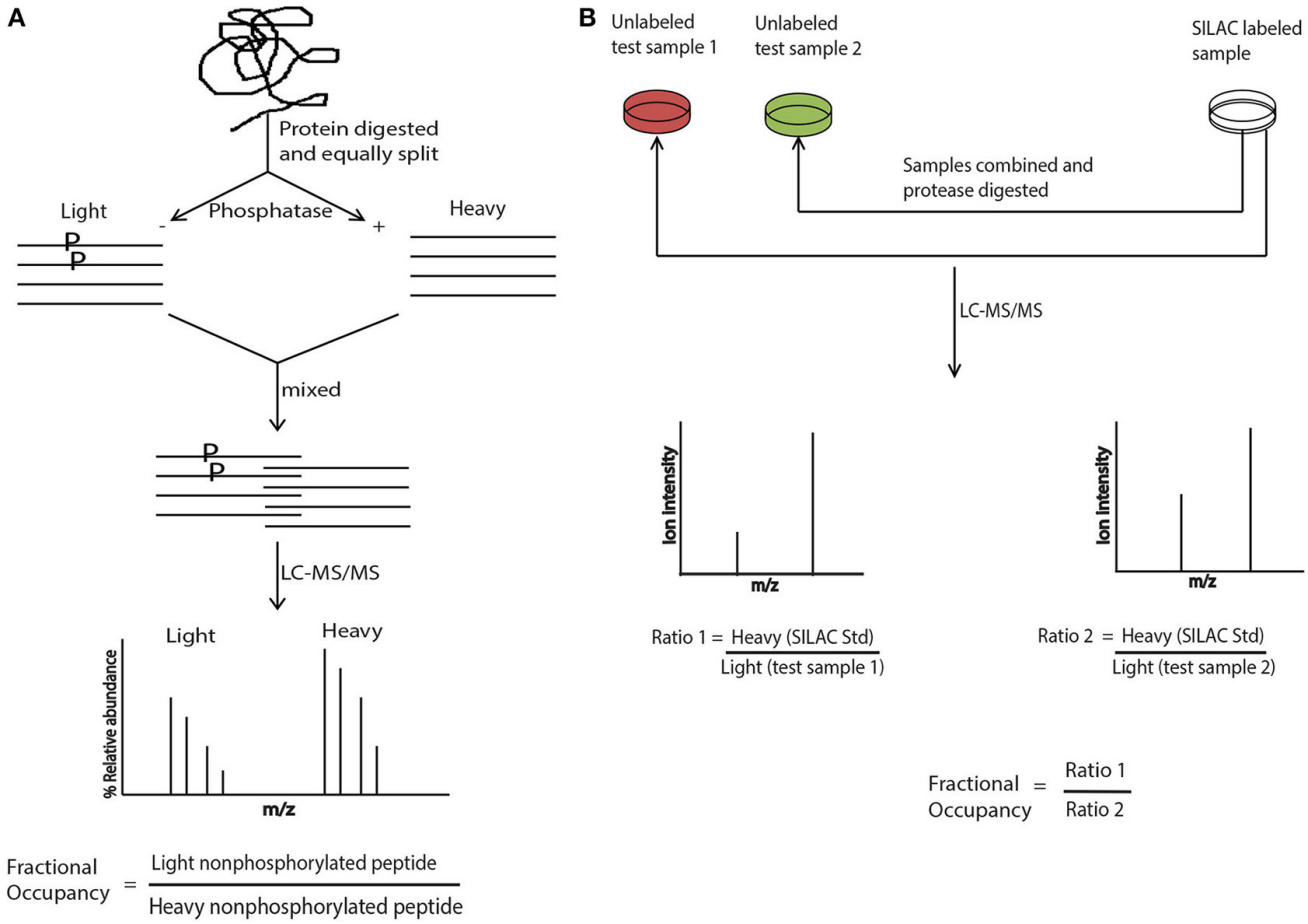
**Absolute quantitation with stable isotope labeling and SILAC. (A)** Protein is digested with a proteaseand divided equally into two aliquots. One aliquot is treated with phosphatase and exposed to heavy isotope and the other aliquot is mock treated and exposed to light label. The two aliquots are combined and subjected to LC-MS/MS analysis. The phosphorylation site occupancy is calculated by taking a ratio of relative abundance of non-phosphorylated light peptide vs. non phosphorylated heavy peptide. **(B)** The SILAC mixture is prepared separately but along with the test samples that are not labeled with any isotope. Subsequently SILAC mixture and the test sample/s are combined, protease digested and subjected to LC-MS/MS analysis. Site occupancy is calculated by taking the “ratio of ratios” where the ratio of the ion intensities of heavy SILAC standard vs. test sample 1 is further divided by the ratio of the ion intensities of heavy SILAC standard vs. test sample 2.

With the present day, state-of-the-art technology and improved methodology we have made significant progress in characterizing phosphorylation and the databank is increasing every day. Nonetheless, another segment of the field; Cys-based PTMs considerably lags behind! The mechanistic insight of Cys-based modifications, in particular, S-nitrosylation and S-palmitoylation, is ever growing, and their involvement in regulating various cellular events is quite plausible. There are few remarkable methods developed over the years to identify the Cys-sites of these modifications with mass spectrometry, like the biotin-switch technique and SNO-Rac for S-nitrosylation (Jaffrey et al., 2001; Forrester et al., 2009) and Acyl-biotin exchange and Acyl-Rac for S-palmitoylation (Drisdel and Green, 2004; Forrester et al., 2011). With these methods serving as benchmark, few studies have attempted relative quantitation of Cys-based modifications in combination with stable isotope labeling (Thompson et al., 2013; Wu et al., 2013), but the absolute quantitation for these modifications would require much groundwork. One possible reason among others which has deterred the progress in this front is the labile nature of these modifications. They are unstable and do not survive the collision energy provided during the MS/MS analysis, whereas phosphorylation, on the contrary, is quite stable. Notwithstanding, direct SNO occupancy measurement has previously been attempted by considering a +29 Kda difference between the modified and unmodified peptide in the ESI-MS mode (Lee et al., 2007; Torta and Bachi, 2012). The broad applicability of such approach is yet to be established, particularly under basal conditions where the SNO occupancy on a given protein is low. Noteworthy, a differential cysteine-reactive tandem mass tag (Cys-TMT) approach was used for the first time under in vivo conditions to show SNO occupancy in the myocardium (Murray et al., 2012), the free and SNO thiols were labeled with light and heavy tags respectively and the SNO occupancy on a given site was calculated as the ratio of the unmodified (light)/modified (heavy) cysteine forms. Consequently, an iodo-acetylTMT (iodo-TMT) sixplex reagent was developed, with the potential benefit of multiplexing the sample (Qiao et al., 2014), this reagent is used so far only in the relative quantitation of SNO-proteins (Qu et al., 2014). These approaches are a significant step towards directing research in site occupancy measurement for Cys-based modifications but might need to be fine-tuned in cases where the abundance of modified-protein or protein *per se* is very low. Thus, looking at the fast pace of the field and the continuous out-flux of ideas from the think-tank, the doorway for site occupancy measurement of Cys-based PTMs is already open but to come up with a foolproof method, would require some sound strategizing.

## Conflict of interest statement

The author declares that the research was conducted in the absence of any commercial or financial relationships that could be construed as a potential conflict of interest.
